# Amino acid substitutions at sugar-recognizing codons confer ABO blood group system-related α1,3 Gal(NAc) transferases with differential enzymatic activity

**DOI:** 10.1038/s41598-018-37515-5

**Published:** 2019-01-29

**Authors:** Emili Cid, Miyako Yamamoto, Fumiichiro Yamamoto

**Affiliations:** 1 Laboratory of Immunohematology and Glycobiology, Josep Carreras Leukaemia Research Institute (IJC), Campus Can Ruti, Camí de les Escoles, Badalona, Barcelona, 08916 Spain; 2 Program of Predictive and Personalized Medicine of Cancer (PMPPC), Institut d’Investigació Germans Trias i Pujol (IGTP), Campus Can Ruti, Camí de les Escoles, Badalona, Barcelona, 08916 Spain

## Abstract

Functional paralogous *ABO*, *GBGT1*, *A3GALT2*, and *GGTA1* genes encode blood group A and B transferases (AT and BT), Forssman glycolipid synthase (FS), isoglobotriaosylceramide synthase (iGb3S), and α1,3-galactosyltransferase (GT), respectively. These glycosyltransferases transfer *N*-acetyl-d-galactosamine (GalNAc) or d-galactose forming an α1,3-glycosidic linkage. However, their acceptor substrates are diverse. Previously, we demonstrated that the amino acids at codons 266 and 268 of human AT/BT are crucial to their distinct sugar specificities, elucidating the molecular genetic basis of the ABO glycosylation polymorphism of clinical importance in transfusion and transplantation medicine. We also prepared *in vitro* mutagenized ATs/BTs having any of 20 possible amino acids at those codons, and showed that those codons determine the transferase activity and sugar specificity. We have expanded structural analysis to include evolutionarily related α1,3-Gal(NAc) transferases. Eukaryotic expression constructs were prepared of AT, FS, iGb3S, and GT, possessing selected tripeptides of AT-specific AlaGlyGly or LeuGlyGly, BT-specific MetGlyAla, FS-specific GlyGlyAla, or iGb3S and GT-specific HisAlaAla, at the codons corresponding to 266–268 of human AT/BT. DNA transfection was performed using appropriate recipient cells existing and newly created, and the appearance of cell surface oligosaccharide antigens was immunologically examined. The results have shown that several tripeptides other than the originals also bestowed transferase activity. However, the repertoire of functional amino acids varied among those transferases, suggesting that structures around those codons differentially affected the interactions between donor nucleotide-sugar and acceptor substrates. It was concluded that different tripeptide sequences at the substrate-binding pocket have contributed to the generation of α1,3-Gal(NAc) transferases with diversified specificities.

## Introduction

α1,3-Gal(NAc) transferases are comprised of blood group A and B transferases (ATs and BTs), Forssman glycolipid synthases (FSs), isoglobotriaosylceramide synthases (iGb3Ss), and α1,3-galactosyltransferases (GTs). Evolutionarily related *ABO* (*A* and *B* alleles), *GBGT1*, *A3GALT2*, and *GGTA1* genes code for those glycosyltransferases, respectively. Blood group ATs and BTs transfer an *N*-acetyl-d-galactosamine (GalNAc) and a d-galactose from donor nucleotide-sugars (UDP-GalNAc and UDP-galactose) to the same acceptor substrate, H substance, to synthesize A and B oligosaccharide antigens, respectively^1,2^. FSs catalyze the last biosynthetic step of pentasaccharide Forssman glycolipid (Gb5) from the precursor globoside (Gb4) by transferring a GalNAc^3^. iGb3Ss and GTs synthesize isoglobotriaosylceramide (iGb3) and α1,3-galactosyl epitope (α-Gal-epitope), respectively. Together with their chemical structures, these molecules are listed in Table 1.

**Table 1:** The list of genes, encoded glycosyltransferases, and catalyzed glycosylation reactions mentioned in the text.

Gene	Glycosyltransferase	Acceptor Substrate(s)	Donor Substrate(s)	Reaction Product(s)
FUT1 (H/h)	α1,2-fucosyltransferase	Galβ1-4GlcNAc- (Core 2 structure)	GDP-fucose	Fucα1-2 Galβ1-4GlcNAc- (Type 2 H)
FUT2 (Se/se)	α1,2-fucosyltransferase	Galβ1-3GlcNAc- (Core 1 structure)	GDP-fucose	Fucα1-2 Galβ1-3GlcNAc- (Type 1 H)
**A allele** **(ABO)** **B allele** **(ABO)**	α1,3-*N*-acetyl-d-galactosaminyltransferase (**AT: A transferase**) α1,3-galactosyltransferase (**BT: B transferase**)	Fucα1-2 Galβ1-3/4GlcNAc- (H substance) Fucα1-2 Galβ1-3/4GlcNAc- (H substance)	UDP-GalNAc UDP-galactose	GalNAcα1-3(Fucα1-2)Galβ1-3/4GlcNAc- (**Blood group A antigen**) Galα1-3(Fucα1-2)Galβ1-3/4GlcNAc- (**Blood group B antigen**)
**A3GALT2**	α1,3-galactosyltransferase (**iGb3S: Isoglobotriaosylceramide synthase**)	Galβ1-4Glcβ1-1′Cer (LacCer: Lactosylceramide)	UDP-galactose	Galα1-3 Galβ1-4Glcβ1-1′Cer (**iGb3: Isoglobotriaosylceramide**)
A4GALT	α1,4-galactosyltransferase	Galβ1-4Glcβ1-1′Cer (LacCer: Lactosylceramide)	UDP-galactose	Galα1-4 Galβ1-4Glcβ1-1′Cer (Gb3: Globotriaosylceramide)
B3GALNT1	β1,3-*N*-acetyl-d-galactosaminyltransferase 1	Galα1-3 Galβ1-4Glcβ1-1′Cer (iGb3: Isoglobotriaosylceramide) Galα1-4 Galβ1-4Glcβ1-1′Cer (Gb3: Globotriaosylceramide)	UDP-galactose	GalNAcβ1-3 Galα1-3 Galβ1-4Glcβ1-1′Cer (iGb4: Isogloboside) GalNAcβ1-3 Galα1-4 Galβ1-4Glcβ1-1′Cer (Gb4: Globoside)
**GBGT1**	α1,3-*N*-acetyl-d-galactosaminyltransferase (**FS: Forssman glycolipid synthase**)	GalNAcβ1-3 Galα1-3 Galβ1-4Glcβ1-1′Cer (iGb4: Isogloboside) GalNAcβ1-3 Galα1-4 Galβ1-4Glcβ1-1′Cer (Gb4: Globoside)	UDP-GalNAc	GalNAcα1-3 GalNAcβ1-3 Galα1-3 Galβ1-4Glcβ1-1′Cer (**iGb5: IsoForssman glycolipid**) GalNAcα1-3GalNAcβ1-3Galα1-4 Galβ1-4Glcβ1-1′Cer (**Gb5: Forssman glycolipid**)
**GGTA1**	α1,3-galactosyltransferase (**GT**)	Galβ1-4GlcNAc- (*N*-acetyllactosamine)	UDP-galactose	Galα1-3 Galβ1-4GlcNAc- (**α-Gal-epitope: α1,3-galactosyl epitope**)

The genes, glycosyltransferases, donor and acceptor substrates, and reaction products mentioned in the text are shown. Evolutionarily related ABO family of genes, their encoded α1,3-Gal(NAc) transferases and their reaction product names are shown in bold.

Being expressed on red blood cells (RBCs), and epithelial and endothelial cells, blood group A and B antigens are of clinical importance in blood transfusion and cell, tissue and organ tranplantations^4,5^. Individuals who do not express A and/or B antigens contain “naturally occurring” anti-A and/or anti-B antibodies in sera, respectively (Landsteiner’s law)^6^. Accordingly, transfusion of ABO-mismatched blood may induce antigen-antibody immune reactions, leading to RBC agglutination followed by complement-mediated hemolysis and release of hemoglobin into blood circulation^7^. If kidneys are overwhelmed by the abundance of lysed RBCs and subsequently fail to filtrate blood efficiently, the recipient may die. Therefore, Landsteiner’s discovery of the ABO system was instrumental to the development of safe blood transfusion practice by avoiding the transfusion in the donor-recipient combinations resulting in RBC agglutination. Together with Rh typing and cross-matching test, forward and reverse ABO typing in pre-transfusion analysis has greatly diminished the incidence of transfusion-related adverse reactions. ABO blood groups were one of the first human characteristics that were demonstrated to inherit through generations, following the Mendelian inheritance^8^, and functional A and B alleles encode AT and BT, respectively, as mentioned above.

In contrast to A and B antigens, the Forssman (FORS1) antigen, isoglobotriaosylceramide (iGb3) and the α-Gal-epitope synthesized by the actions of FS, iGb3S, and GT, respectively, are not so well recognized. However, those glycans are also important and carry out their own functions of biological and medical significance. For instance, FORS1 is the blood group antigen responsible of FORS system^9,10^. The frequency of individuals expressing the FORS1 antigen (presenting what was called A_pae_ phenotype) is extremely low in the human population (rs375748588 SNP, MAF/Minor Allele Count: T = 0.000008/1 (ExAC), T = 0.00008/1 (GO-ESP), T = 0.00005/6 (TOPMED)). Nonetheless, when the FORS1-negative recipient has anti-FORS1 antibodies, FORS1-positive RBC transfusion may induce RBC agglutination^9,11–14^. Therefore, such a combination of blood transfusion should be avoided. iGb3 was implicated in the intra-thymus selection of CD1d-restricted invariant natural killer T (iNKT) cells^15^ and their activation in the periphery^16,17^. Although later studies have cast doubt on the hypothesis that iGb3 is the unique self-antigen required for iNKT cells^18–20^, it is still possible that autologous glycolipids that are structurally related to iGb3 may contribute to the positive iNKT cell selection. Lastly, humans do not express α-Gal-epitope but possess anti-Gal antibodies against this glycan instead. Actually, anti-Gal is one of the most abundant natural antibodies in humans, consisting nearly 1% of immunoglobulins^21^. Anti-Gal IgM and IgG antibodies may elicit acute rejection of xenografted organs expressing this epitope^22^, and anti-Gal IgE may cause allergies to meat and therapeutic monoclonal antibodies presenting the epitope^23^. Aberrant expression of α-Gal-epitope or of antigens mimicking it may result in autoimmune reactions such as in Graves’ disease^24^. In summary, all of those genes/glycosyltransferases/glycans have relevance to normal physiology and/or disease pathology. Accordingly, it is essential to better understand the molecular structural mechanisms that have generated this family of α1,3-Gal(NAc) transferases with different substrate specificities.

α1,3-Gal(NAc) transferases and their genes are present in a variety of vertebrate species, and the repertoire is species-dependent^25^. For instance, humans may have functional AT and/or BT, depending on the polymorphic status at the *ABO* genetic locus, but they carry none of the functional *GBGT1*, *A3GALT2*, or *GGTA1* genes nor exhibit any of FS, iGb3S, or GT activities^26–29^. However, exceptions exist. Rare FORS1-positive individuals possess a dominant missense mutation in the *GBGT1* gene, resulting in the acquisition of FS activity and the appearance of FORS1 antigen^9,30^. Birds are another example of species-dependent repertoire. They may maintain functional *GBGT1* gene-encoded FS, but lack any other α1,3-Gal(NAc) transferase genes/proteins. Contrastingly, mice may have functional *ABO*, *GBGT1*, *A3GALT2*, and *GGTA1* genes and exhibit those four transferase activities. This disparity of species-dependent presence/absence of functional genes/transferases follows the birth and death model of gene evolution^31,32^. We have recently proposed a theory that chromosomal rearrangements are responsible, at least partially, for the formation of diverged gene distribution^33^.

Blood group AT and BT are different only by four amino acids at codons 176, 235, 266, and 268^2,34^. They are arginine (Arg), glycine (Gly), leucine (Leu), and Gly in AT, and Gly, serine (Ser), methionine (Met), and alanine (Ala) in BT. We prepared 14 AT-BT chimeras that were different at those 4 positions, having either amino acid of AT or BT, transfected DNA from those constructs into human cervical cancer HeLa cells expressing H substance, and immunologically examined the appearance of A and B antigens^35^. We found that the amino acid substitutions at codons 266 and 268 are critical to confer the distinct sugar specificities of those transferases, whereas those at 235 and 176 exhibited slight and no effects, respectively. We later prepared various amino acid substitution constructs at codons 266, 268, and nearby positions, and performed similar experiments^25,36^. The results revealed that the size and charge of the side chain of those amino acids determine both sugar specificity and transferase activity. *In vitro* mutagenesis studies from other laboratories^37,38^, three-dimensional structural determination of GT and AT/BT with and without substrates^39–42^, as well as structural modeling of α1,3-Gal(NAc) transferases^9,43^, have also contributed to the better understanding of the structural basis of α1,3-Gal(NAc) transferase functions.

In the present study we have expanded *in vitro* mutagenesis investigation to include other α1,3-Gal(NAc) transferases than AT/BT, namely, FS, iGb3S, and GT. Because of the well-characterized functional significance of codons 266 to 268 of human AT/BT, we have focused our research on amino acid substitutions at the corresponding codons of other transferases. Here, we report that they are also critical for their activities and differentially influence the donor and acceptor substrate specificities.

## Results

### Immunocytochemical detection using monoclonal anti-glycan antibodies after DNA transfection experiments of expression constructs revealed differential tripeptide effects on α1,3-Gal(NAc) transferase activities and specificities

HeLa(FUT2) cells were previously created from HeLa cells to achieve enhanced detection sensitivity of AT and BT activities by retrovirally transducing human *FUT2* gene cDNA encoding α1,2-fucosyltransferase that catalyzes the last biosynthetic step of H substance, the acceptor substrate for AT and BT^44,45^. COS1(B3GALNT1) cells were similarly generated from African green monkey kidney COS1 cells by the modular expression of human *B3GALNT1* gene cDNA-encoded β1,3-*N*-acetyl-d-galactosaminyltransferase 1 to produce increased Gb4, the acceptor substrate for FS^46^. In this study, we successfully generated Chinese hamster ovary CHO(B3GALNT1 + GBGT1) cells that express human *B3GALNT1* and mouse *GBGT1* gene cDNAs to facilitate the detection of iGb3S activity. Together with COS1 cells to detect the GT activity, those cells were used as recipients for DNA transfection of the successfully prepared eukaryotic expression constructs encoding AT, FS, iGb3S, and GT, having either of AlaGlyGly, GlyGlyAla, HisAlaAla, LeuGlyGly, or MetGlyAla tripeptide sequence at codons corresponding to 266–268 of human AT/BT.

Results of immunocytochemical detection are shown in Table 2. We have found that several constructs having tripeptide sequences that were different from the original ones also exhibited some glycosyltransferase activity as demonstrated by the cell surface appearance of specific glycan antigens. As previously shown, the original human AT construct with LeuGlyGly and the substitution construct with AlaGlyGly exhibited strong AT activity whereas the construct with MetGlyAla exhibited strong BT activity^25^. The AT construct with GlyGlyAla exhibited both AT and BT activities^47^. Additionally, the present results also showed that the human AT with HisAlaAla exhibited BT activity. The mouse FS with the AlaGlyGly tripeptide exhibited FS activity as strong as the original mouse FS with GlyGlyAla. Similarly, in addition to the original iGb3S with HisAlaAla, iGb3Ss with AlaGlyGly, GlyGlyAla, LeuGlyGly, or MetGlyAla also exhibited the iGb3S activity although its strength varied widely. Furthermore, the original mouse GT with HisAlaAla expressed strong GT activity, whereas the mutants with GlyGlyAla or MetGlyAla presented weak activity. Interestingly, transferase activities not corresponding to the enzyme were also observed. In addition to AT with GlyGlyAla, whose FS activity has recently been reported^46^, we have also observed FS activity of AT with AlaGlyGly.

**Table 2:** Immunocytochemical detection of AT/BT, FS, iGb3S, and GT activities.

Set	I				II				III				IV				V			
Cell	HeLa(FUT2)				HeLa(FUT2)				COS1(B3GALNT1)				CHO (B3GALNT1 + GBGT1)				COS1			
Antigen	A				B				FORS1				FORS1				α-Gal-epitope			
Exp.	1	2	3	AT	1	2	3	BT	1	2	3	FS	1	2	3	iGb3S	1	2	3	GT
**H_ABO-A**																				
AGG	100.0	140.6	95.0	5+	0.0	0.0	0.0	—	16.7	15.0	15.7	3+	0.0	0.0	0.0	—	0.0	0.0	0.0	—
GGA	87.5	109.4	87.5	5+	44.0	35.7	36.7	4+	26.7	26.7	25.0	3+	0.0	0.0	0.0	—	0.0	0.0	0.0	—
HAA	0.3	0.6	0.5	+/—	48.0	39.3	40.0	4+	0.0	0.0	0.0	—	0.0	0.0	0.0	—	0.0	0.0	0.0	—
*LGG*	100.0	100.0	100.0	5+	0.0	0.0	0.0	—	0.0	0.0	0.0	—	0.0	0.0	0.0	—	0.0	0.0	0.0	—
MGA	2.5	3.1	3.0	+	100.0	100.0	100.0	5+	0.0	0.0	0.0	—	0.0	0.0	0.0	—	0.0	0.0	0.0	—
**M_GBGT1**																				
AGG	0.5	0.7	0.4	+/−	3.2	2.1	1.7	+	90.0	83.3	83.3	5 +	0.0	0.0	0.0	—	0.0	0.0	0.0	—
*GGA*	3.8	3.4	3.3	+	0.8	1.1	0.3	+/−	100.0	100.0	100.0	5+	0.0	0.0	0.0	—	0.0	0.0	0.0	—
HAA	0.6	0.9	0.4	+/−	0.1	0.4	2.3	+/−	0.0	1.0	0.0	+/−	0.0	0.0	0.0	—	0.0	0.0	0.0	—
LGG	2.0	3.1	2.3	+	2.0	3.6	2.0	+	0.7	0.0	0.0	+/−	0.0	0.0	0.0	—	0.0	0.0	0.0	—
MGA	1.0	2.2	1.5	+/−	1.6	1.4	1.7	+/−	0.0	0.0	0.0	—	0.0	0.0	0.0	—	0.0	0.0	0.0	—
**R_A3GALT2**																				
AGG	0.0	0.0	0.0	—	0.0	0.0	0.0	—	0.0	0.0	0.0	—	6.0	6.7	5.0	2+	0.0	0.0	0.0	—
GGA	0.0	0.0	0.0	—	0.0	0.0	0.0	—	0.0	0.0	0.0	—	5.0	5.6	6.0	2+	0.0	0.0	0.0	—
*HAA*	1.8	0.9	0.6	+/−	1.6	0.1	0.1	+/−	0.0	0.0	0.0	—	100.0	100.0	100.0	5+	4.2	2.5	5.0	+
LGG	0.3	0.0	0.1	—	0.6	0.5	0.3	+/−	0.0	0.0	0.0	—	22.0	22.2	20.0	3+	0.5	0.2	0.6	+/−
MGA	0.1	0.1	0.8	+/−	0.3	0.2	0.1	+/−	0.0	0.0	0.0	—	14.0	22.2	20.0	3+	0.0	0.3	0.0	—
**M_GGTA1**																				
AGG	0.0	0.1	0.3	—	0.2	0.0	0.3	+/−	0.0	0.0	0.0	—	0.8	0.0	0.0	+/−	0.2	0.5	0.6	+/−
GGA	0.0	0.0	0.0	—	0.0	0.0	0.0	—	0.0	0.0	0.0	—	0.2	0.2	0.2	+/−	16.7	16.7	16.0	3+
*HAA*	0.0	0.0	0.1	—	0.0	0.0	0.0	—	0.0	0.0	0.0	—	0.3	0.0	0.6	+/−	100.0	100.0	100.0	5+
LGG	0.0	0.0	0.0	—	0.1	0.0	0.0	—	0.0	0.0	0.0	—	0.2	0.2	0.4	+/−	1.7	0.8	2.0	+/−
MGA	0.0	0.2	0.0	—	0.0	0.0	0.0	—	0.0	0.0	0.0	—	0.6	0.7	0.6	+/−	11.7	10.0	10.0	2+

Five sets (I–V) of experiments were performed in triplicates (Exp. 1–3) using 4 cell types and 4 antigens analyzed. The original gene names and the tripeptide sequences at the codons corresponding to 266–268 of human AT/BT are listed in the leftmost column: H_ABO-A, M_GBGT1, R_A3GALT2, and M_GGTA1 for the *A* allele of human *ABO* gene, mouse *GBGT1* gene, rat *A3GALT2* gene, and mouse *GGTA1* gene, respectively. The tripeptides are abbreviated: AGG (AlaGlyGly), GGA (GlyGlyAla), HAA (HisAlaAla), LGG (LeuGlyGly), and MGA (MetGlyAla). The original tripeptide sequences are shown in *italic*. The antigen positive cell percentages were re-calculated so that those of the original positive control constructs become 100%. The deduced AT, BT, FS, iGb3S, and GT activities are shown semi-quantitatively (-: no, +/−: scarce, + : very weak, 2 + : weak, 3 + : moderate, 4 + : strong, 5 + : very strong activity).

### Quantification by flow cytometry confirmed differential effects of tripeptides on substrate specificities of *in vitro* mutagenized α1,3-Gal(NAc) transferases

**Figure 1:**
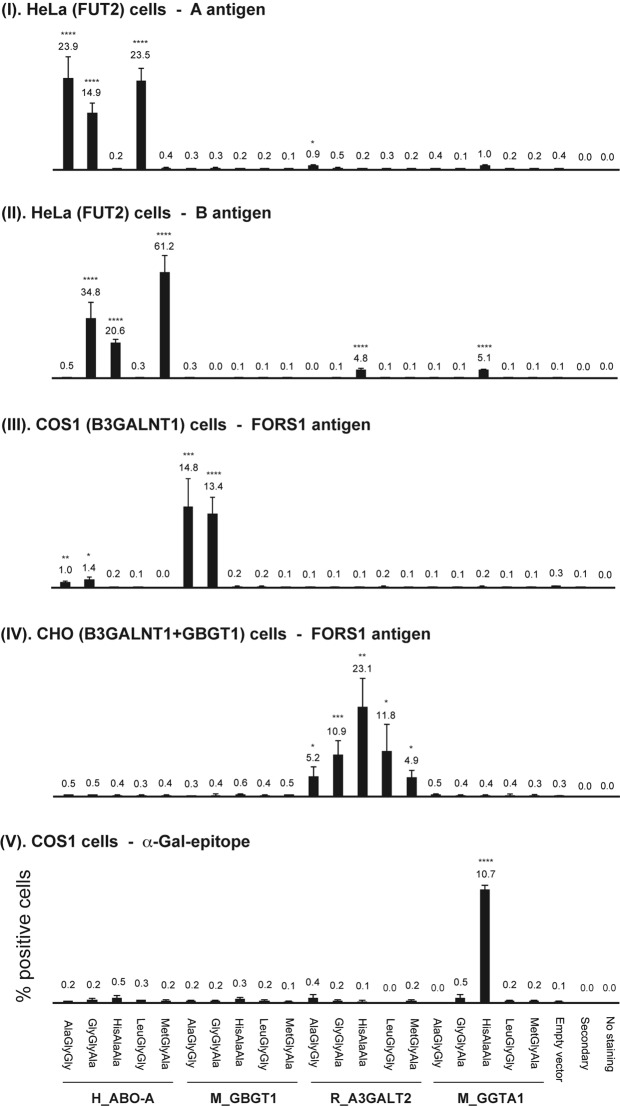
Expression of glycan antigens after DNA transfection of the 20 expression constructs into appropriate recipient cells analyzed by flow cytometry. After DNA transfection of cells with a variety of expression constructs and a vector expressing GFP or mRFP, cells were immunostained using anti-glycan antibodies, followed by flow cytometry. Average ( + SEM) values of the antigen-positive cell percentages (without adjustment to the original constructs) were calculated and are depicted as bar graphs. The construct names are shown at the bottom of the figure. Asterisks indicate the p values resulting from Student’s t-tests comparing each group with the empty vector control: ****P ≤ 0.0001, ***P ≤ 0.005, **P ≤ 0.01, and *P ≤ 0.05.

**Figure 2:**
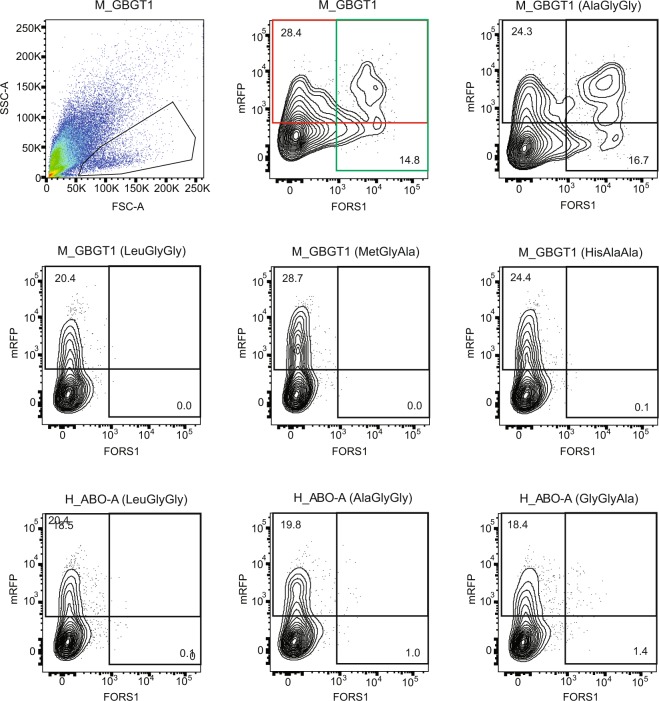
COS1(B3GALNT1) cells transfected with M_GBGT1(AlaGlyGly) or H_ABO-A(AlaGlyGly) expressed FORS1 antigen. Cells transfected with selected expression constructs were analyzed for FORS1 glycan expression by detecting AF488 fluorescence at 530 nm, together with the expression of mRFP fluorescence at 575 nm. On the top row, the first two figures correspond to the transfection experiments of the original M_GBGT1 construct. Single viable cell population was selected based on forward (FSC-A) and side (SSC-A) scattering amplitudes on the left. Events were plotted in pseudocolor with red representing higher frequency and dark blue the lowest, and the gate set to select cell population for analysis is indicated. The same selection was maintained for all the other samples. Next to it, a contour plot shows the mRFP fluorescence intensity, used as transfection control, on the y-axis, and the AF488 fluorescence corresponding to FORS1 on the x-axis. Both axes are logarithmic in scale. The upper red rectangle represents the gate containing the mRFP positive cells and the percentage of mRFP + cells is also indicated at the upper left corner. The green rectangle corresponds to the gate containing the AF488-positive cells, and the percentage is indicated at the lower right corner. The other graphs show the results from flow cytometry of cells transfected with other constructs.

**Figure 3:**
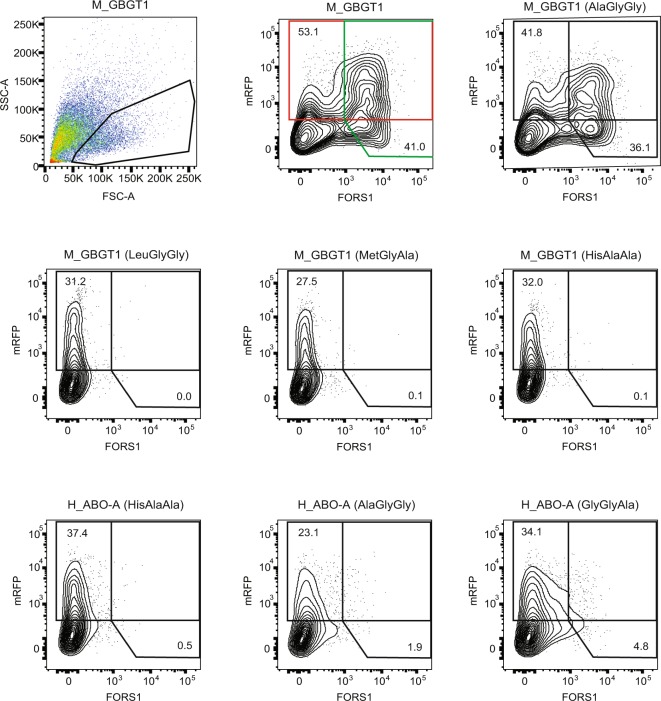
FORS1 antigen expression was also observed of COS1(B3GALNT1) cells transfected with M_GBGT1(AlaGlyGly) or H_ABO-A(AlaGlyGly) using Lipofectamine 3000 and immunostained 2 days after DNA transfection. Whereas Fig. 2 shows the flow cytometry results of cells transfected with selected expression constructs using Lipofectamine 2000 and analyzed for FORS1 glycan expression 20 hours after DNA transfection, this figure shows those of cells transfected using Lipofectamine 3000 and analyzed 2 days after DNA transfection, employing the same immunostaining and flow cytometry protocols.

In order to obtain more quantitative data, DNA transfected cells were also analyzed by flow cytometry. Results are shown in a bar graph in Fig. 1. Flow cytometry experiments have presented similar results to immunocytochemistry although there are minor discrepancies especially for those constructs that exhibited weak antigen expression. Human AT constructs with AlaGlyGly, GlyGlyAla, HisAlaAla, LeuGlyGly, or MetGlyAla exhibited AT, AT/BT, BT, AT, or BT activity, respectively. Mouse FSs with AlaGlyGly or GlyGlyAla exhibited FS activity whereas mouse FSs with HisAlaAla, LeuGlyGly, or MetGlyAla did not. Contrastingly, iGb3Ss with AlaGlyGly, GlyGlyAla, LeuGlyGly, or MetGlyAla, in addition to the original HisAlaAla, all exhibited the iGb3S activity although its strength varied. Strong GT activity was detected with the original GT with HisAlaAla and weak GT activity with GlyGlyAla. In addition, we also observed FS activity of human ATs with AlaGlyGly or GlyGlyAla. These two flow cytometry results are shown in Figs 2 and 3, together with the data obtained with several other constructs. Figure 2 represents the results obtained 1 day after DNA transfection using Lipofectamine 2000 reagent, whereas Fig. 3 represents those obtained 2 days after DNA transfection using Lipofectamine 3000. Both results exhibited a similar tendency although higher numbers of FORS1-positive cells were observed with several constructs in the latter experiments.

## Discussion

Paralogous genes have been generated through gene duplications and following divergence. In addition to entire genes, gene portions have also been duplicated. If structural gene sequences were linked to different promoter/enhancer/insulator elements, the expression profile may have been altered. If certain exon(s) were duplicated and connected to different preceding or subsequent exon(s), newly created gene-encoded proteins would lose original structures and/or functions in the majority of cases. However, at rare occasions during the long history of evolution, some may have acquired novel structures and/or functions instead. Phylogenetic analyses of α1,3-Gal(NAc) transferase genes have previously shown that a common ancestral gene was duplicated and then they were separated into the *ABO/GBGT1* gene lineage and the *A3GALT2/GGTA1* gene lineage, followed by later separations, the former into the *ABO* and *GBGT1* genes and the latter into the *A3GALT2* and *GGTA1* genes^25,31^. α1,3-Gal(NAc) transferases are, therefore, an example of evolutionary divergence by acquisition of distinct donor and/or acceptor substrate specificities. In addition to *ABO*, *GBGT1*, *A3GALT2*, and *GGTA1* genes, another homologous gene named *GLT6D1* (glycosyltransferase 6 domain containing 1) has been identified. Single nucleotide polymorphism (SNP) markers at this genetic locus were associated with the susceptibility to periodontitis^48^. However, because no transferase activity has been reported, *GLT6D1*-encoded protein was excluded from the current analysis.

Among glycosyltransferases involved in the biosynthesis of other blood group glycan antigens than A/B and FORS1, a single amino acid at codon 111 in the hypervariable stem domain of *FUT3* gene-encoded α1,3/1,4-fucosyltransferase specifying Lewis phenotype was previously shown to determine the usage of type 1 or type 2 acceptor substrates^49^. In addition, whereas the consensus human *A4GALT* gene-encoded α1,4-galactosyltransferase (Gb3S) may synthesize the P^k^ and P1 antigens of P1PK blood group, its p.Q211E variant possessing the glutamine to glutamic acid substitution at codon 211 was shown to synthesize all three antigens: P^k^, P1 and NOR^50^. In the present study, we have focused our attention on the catalytic domains of α1,3-Gal(NAc) transferases. More specifically, we have investigated the effects of amino acid substitutions of human AT, mouse FS, rat iGb3S, and mouse GT at the tripeptide sequences corresponding to codons 266 to 268 of the human AT/BT on enzymatic specificity. In addition that those amino acids are critical to different sugar specificities of AT and BT^35,36^, they were shown to physically interact with donor nucleotide-sugars^39–42^. Our results have shown that several other tripeptides than the originals also bestowed transferase activity although the repertoire of functional amino acids varied among those transferases. Among those four α1,3-Gal(NAc) transferases, the donor sugars are α-GalNAc in AT and FS and α-galactose in BT, iGb3S, and GT, whereas the acceptor sugar is β-GalNAc for FS and β-galactose for AT/BT, iGb3S, and GT. The fact that human ATs with AlaGlyGly or GlyGlyAla also exhibited some FS activity suggests that ATs may have less stringent acceptor substrate specificity. The results also manifested that the structures around those tripeptide codons might impact differentially on the interaction with acceptor substrates, in addition to donor nucleotide-sugar substrates.

Quite recently, we have demonstrated that GlyGlyAla is vital to the mouse *GBGT1* gene-encoded FS activity because the substitution by either AT-specific LeuGlyGly or BT-specific MetGlyAla abolished the FS activity^46^. We have also found that mouse *ABO* gene-encoded protein, which was previously shown to exhibit the *cis*-AB transferase characteristics to synthesize both A and B antigens, contains the GlyGlyAla tripeptide and exhibits the FS activity. Furthermore, the replacement of this GlyGlyAla sequence of the mouse *cis*-AB transferase with LeuGlyGly or MetGlyAla abolished the FS activity while retaining AT or BT activity, respectively. Moreover, the replacement of the LeuGlyGly sequence of human AT with GlyGlyAla has conferred some, though not as strong, FS activity. Here we have found that human AT with AlaGlyGly also expresses FS activity. As opposed to GlyGlyAla, which is rarely present in the *ABO* genes, the AlaGlyGly tripeptide is frequent in ATs among vertebrate species, excluding primates where LeuGlyGly is predominant^25,51^. Therefore, those ATs may also exhibit FS activity under certain conditions where appropriate acceptor substrates are present. If this is the case, two genetic loci, *GBGT1* and *ABO*, specify FORS1 expression. Accordingly, the genetic determination of Forssman positivity/negativity may be confounded in those species.

In this study we have employed glycan detection by immunocytochemistry and flow cytometry. As opposed to immunocytochemistry where signal could be amplified by the peroxidase enzymatic reaction, flow cytometry signal was not so strong. Normalizing the FORS1 + cell percentage of M_GBGT1(GlyGlyAla) to be 100.0, those for M_GBGT1(AlaGlyGly), H_ABO-A(AlaGlyGly), and H_ABO-A(GlyGlyAla) were calculated to be (90.0, 83.3, 83.3), (16.7, 15.0, 15.7), and (26.7, 26.7, 25.0), respectively, in 3 separate immunocytochemistry experiments (Table 2). On the other hand, the flow cytometry results in Figs 1–3 are shown without normalization. However, those values could be normalized to become 100.0, 104.5, 7.5, and 10.4 (Fig. 1), 100.0, 117.3, 8.5, and 9.6 (Fig. 2), and 100.0, 111.5, 11.0, and 17.6 (Fig. 3), respectively. Therefore, they exhibited similar tendencies irrespective of different transfection reagents (Lipofectamine 2000 vs. 3000) and different incubation periods before immunostaining (1 day vs. 2 days). They were also similar to the averages of 3 immunocytochemistry results of 100.0, 85.5, 15.8, and 26.1 except that the M_GBGT1(AlaGlyGly) values were higher in flow cytometry than in cytochemistry.

Regarding negative results, the interpretation may require sufficient caution. In case of blood group A and B antigens, monoclonal antibodies that discriminate those antigens are available. Therefore, the conversion of sugar specificity between GalNAc and galactose may be easily detected, using those antibodies. However, due to the absence of appropriate antibodies, the immunological detection of the reaction products may be difficult for FS, iGb3S, or GT if the sugar specificity is altered. There also remains a possibility that novel glycan structures are synthesized by the alterations in substrate specificity of those enzymes. In this study we have restricted the usage of antibodies only to those against blood group A, B, FORS1, and α-Gal epitope in spite of the fact that many other anti-glycan antibodies, as well as lectins, were available to characterize glycans that might have appeared. We believe that future studies employing mass spectrometry will conclusively determine the chemical structures of those enzymatic reaction products and provide answers to those interesting hypotheses.

## Methods

### Comparison of deduced amino acid sequences of α1,3-Gal(NAc) transferase cDNAs used in the study

Several distinct splicing patterns have been observed of messenger RNAs encoding ATs/BTs, FSs, iGb3Ss, or GTs. Therefore, the deduced amino acid sequences of the selected cDNAs used to prepare the eukaryotic expression constructs of α1,3-Gal(NAc) transferases were compared and aligned, using the ClustralW program of MEGA5 software^52^. Results are shown in Supplemental Fig. 1.

### Construction of *in vitro* mutagenized amino acid substitution constructs of α1,3-Gal(NAc) transferases

Human AT and BT, mouse FS, rat iGb3S, and mouse GT possess the following tripeptide sequences: LeuGlyGly, MetGlyAla, GlyGlyAla, HisAlaAla, and HisAlaAla, respectively, at codons corresponding to 266–268 of the human AT/BT. They have been conserved among a variety of vertebrates except ATs, for which both LeuGlyGly and AlaGlyGly are frequent^25^. Therefore, we decided to analyze the enzymatic activities of AT, FS, iGb3S, and GT, having either of AlaGlyGly, GlyGlyAla, HisAlaAla, LeuGlyGly, or MetGlyAla tripeptide sequence. The total amounted to 20 expression constructs.

Eight constructs were previously prepared and available: H_ABO-A, H_ABO-A(MetGlyAla), H_ABO-A(AlaGlyGly), and H_ABO-A(GlyGlyAla)^25^, M_GBGT1, M_GBGT1(LeuGlyGly), and M_GBGT1(MetGlyAla)^46^, and M_GGTA1. The last construct previously named as mouse GT contained Ggta1-202 transcript cDNA cloned from mouse testis embryonal carcinoma F-9 cells^53^. Therefore, only the remaining 12 constructs were prepared anew.

Firstly, we performed polymerase chain reaction (PCR) using appropriate primers and rat A3galt2-201 cDNA cloned in pCMV SPORT6 vector (OriGene) as template, subcloned the amplified DNA fragment into pSG5 vector (Stratagene), obtaining the rat iGb3S construct: R_A3GALT2. Then, we prepared the following 11 constructs by *in vitro* mutagenesis: H_ABO-A(HisAlaAla), M_GBGT1(AlaGlyGly), M_GBGT1(HisAlaAla), R_A3GALT2(AlaGlyGly), R_A3GALT2(GlyGlyAla), R_A3GALT2(LeuGlyGly), R_A3GALT2(MetGlyAla), M_GGTA1(AlaGlyGly), M_GGTA1(GlyGlyAla), M_GGTA1(LeuGlyGly), and M_GGTA1(MetGlyAla).

We introduced amino acid substitutions by the 2-round primer-mediated PCR strategy using primers with nucleotide substitutions^25,54^. The PCR reaction products were digested with *EcoRI* and *BamH1*, ligated with similarly digested, and additionally dephosphorylated, pSG5 vector, and used to transform frozen competent TOP10 strain of *Escherichia coli* bacteria. Plasmid DNA was prepared from several clones, and the cDNA inserts were sequenced. The constructs without any additional unintended non-synonymous mutations were selected for subsequent experiments.

### Generation of CHO(B3GALNT1+GBGT1) cells

To easily detect the iGb3S activity, we generated CHO(B3GALNT1 + GBGT1), a derivative cell line of Chinese hamster ovary (CHO) cells, by transducing cells with human *B3GALNT1* gene cDNA and mouse *GBGT1* gene cDNA under a viral promoter to express β1,3-*N*-acetyl-d-galactosaminyltransferase 1 and FS, respectively. When iGb3 is synthesized by functional iGb3S in those cells, it undergoes sequential glycosylation to become isogloboside (iGb4) by β1,3-*N*-acetyl-d-galactosaminyltransferase 1 and then to isoForssman (iGb5) by FS. Clone FOM-1 rat anti-FORS1 monoclonal antibody recognizes FORS1 antigen on both Gb5 and iGb5. Therefore, the iGb3S activity is indirectly measured by immunologically detecting FORS1 on iGb5 appearing on the DNA-transfected CHO(B3GALNT1 + GBGT1) cells.

We employed the glycan customization technology by modular expression of glycosyltransferases, which we developed in our laboratory^44^. We inserted mouse FS cDNA into the pMigR1r retroviral vector that contains the internal ribosome entry site (IRES) element followed by TagRFP647 protein cDNA, and prepared the pMigR1r-GBGT1 construct. The pMigR1b-B3GALNT1 construct, which was previously used to prepare COS1(B3GALNT1) cells, and the newly prepared pMigR1r-GBGT1 construct were separately transfected into GP2-293 packaging cells, together with pVSV-G vector encoding the vesicular stomatitis virus G protein. Then GP2-293 cell culture media containing viral particles were recovered and filtered. CHO cells were infected with the two different viral particles and allowed to grow to confluence. After detaching them with trypsin/EDTA and re-suspended in cell culture media, cells expressing both the coupled blue and far-red emitting fluorescent proteins (BFP and RFP647) were FACS-sorted to generate CHO(B3GALNT1 + GBGT1) cells. The selected cells were expanded and used as a recipient of DNA transfection using 20 expression constructs of α1,3-Gal(NAc) transferases.

### DNA transfection experiments

For immunocytochemistry experiments DNA transfection was performed using Lipofectamine 3000 reagents, following the manufacturer’s protocol (Thermo Fisher Scientific). In order to normalize transfection efficiency, a fixed amount of DNA from pEGFP construct expressing enhanced green fluorescent protein (GFP) was co-transfected. A total of 100 transfection experiments were performed with 20 expression constructs and 4 different recipient cell types: HeLa(FUT2) cells in duplicates to separately detect AT and BT activities, and COS1(B3GALNT1), CHO(B3GALNT1 + GBGT1), and COS1 cells to detect FS, iGb3S, and GT activities, respectively. The entire experiments were repeated in triplicates.

For flow cytometry experiments, DNA from individual expression constructs was co-transfected with either pEGFP plasmid DNA encoding GFP to HeLa(FUT2) cells or pLL3.7-mRFP DNA encoding monomeric RFP to COS1, COS1(B3GALNT1) and CHO(B3GALNT1 + GBGT1) cells. This was necessary to avoid interferences with fluorescent proteins expressed by retroviral vector(s) introduced to generate the recipient cells, as well as differences in the immunological methods of antigen detection. The experiments were done in triplicate. In the majority of DNA transfection for flow cytometry, Lipofectamine 2000 reagents (Thermo Fisher Scientific) were used, whereas several selected constructs were also transfected to COS1(B3GALNT1) cells using Lipofectamine 3000 for comparison.

### Immunocytochemical detection of blood group A and B antigens, FORS1 antigen, and α-Gal-epitope

Three days after DNA transfection and just before cell fixation with 4% paraformaldehyde, GFP-positive cells were counted under fluorescence microscopy. After fixation, cells were washed, air-dried, and immunostained using appropriate antibodies. The mixture of anti-A murine IgM monoclonal antibodies, Anti-A (Anti-ABO1) BioClone (reference number 711228), and the mixture of anti-B murine IgM monoclonal antibodies, Anti-B (Anti-ABO2) BioClone (reference number 711328) from Ortho Clinical Diagnostics, were separately used to detect the A and B antigens expressed on DNA-transfected HeLa(FUT2) cells, respectively. Clone FOM-1 anti-Forssman rat IgM monoclonal antibody (BMA Biomedicals) was used to identify FORS1 on Forssman and isoForssman glycolipids, Gb5 and iGb5, appeared on DNA-transfected COS1(B3GALNT1) and CHO(B3GALNT1 + GBGT1) cells, respectively. The expression of α-Gal-epitopes was monitored using the mouse-human chimeric antibody consisting of the variable domain of clone M86 mouse monoclonal antibody against the α-Gal-epitope linked to the constant region of human IgG1 (Absolute Antibody).

Cells were firstly incubated with a primary antibody, washed, and next incubated with an appropriate biotinylated goat secondary antibody against mouse IgM (for A and B antigen detection), rat Ig(M + G) (for FORS1), or human IgG (for α-Gal-epitope). Then, Avidin: Biotinylated enzyme Complex (ABC) technique was employed to visualize detection by the peroxidase color development, using the Vectastain ABC kits and 3,3′-Diaminobenzidine (DAB) substrate (Vector Laboratories). Positive cell numbers were counted under the microscope.

### Flow cytometry

For flow cytometry experiments, cells were detached from culture plates with EDTA, washed, and then subjected to immunostaining twenty hours after DNA transfection in the majority of experiments. For flow cytometry of COS1(B3GALNT1) cells transfected using Lipofectamine 3000, immunostaining was performed 2 days after transfection. DNA transfected HeLa(FUT2) cells were first incubated with anti-A murine monoclonal antibodies or anti-B murine monoclonal antibodies followed by Alexa Fluor 647 (AF647)-conjugated goat anti-mouse IgM (µ chain specific) secondary antibody (Thermo Fisher Scientific). Then, the cells were subjected to flow cytometry, using the BD LSRFortessa Analyzer (BD Biosciences). The GFP fluorescence was measured at 530 nm to identify co-transfected GFP expression and to determine the transfection efficiency. The A and B antigen expression was measured by the AF647 fluorescence at 660 nm. DNA transfected COS1(B3GALNT1) and CHO(B3GALNT1 + GBGT1) cells were initially incubated with clone FOM-1 anti-FORS1 rat IgM monoclonal antibody and then with Alexa Fluor 488 (AF488)-conjugated goat anti-rat IgM (µ chain specific) secondary antibody (Thermo Fisher Scientific). The cells were then subjected to flow cytometry. The mRFP fluorescence was measured at 575 nm to identify co-transfected RFP expression, and the AF488 fluorescence was detected at 530 nm to measure the FORS1 expression. DNA transfected COS1 cells were immunostained with the mouse-human chimeric antibody against the α-Gal-epitope, biotinylated goat anti-human IgG (Vector Laboratories), and then with AF488-conjugated Streptavidin (Thermo Fisher Scientific). Fluorescence of the mRFP and AF488 was measured at 575 nm and 530 nm, respectively.

In additional experiments the constructs exhibiting positive results were repeatedly analyzed, together with positive and negative controls. The averages and standard error of the mean (SEM) values were determined, and statistical significance was assessed.

## Supplementary information

Supplemental Figure 1

## References

[1] Yamamoto, F., Cid, E., Yamamoto, M. & Blancher, A. ABO research in the modern era of genomics. *Transfus Med Rev* **26**, 103–118, S0887-7963(11)00079-4 (2012). 10.1016/j.tmrv.2011.08.002 21945157

[2] Yamamoto F, Clausen H, White T, Marken J, Hakomori S (1990). Molecular genetic basis of the histo-blood group ABO system. Nature.

[3] Siddiqui B, Hakomori S (1971). A revised structure for the Forssman glycolipid hapten. J Biol Chem.

[4] Harmening, D. *Modern blood banking and transfusion practices*. 2nd edn, (F.A. Davis, 1989).

[5] Anderson, K. C. & Ness, P. M. *Scientific Basis of Transfusion Medicine: Implications for Clinical Practice*. 2nd edn, (Saunders, W. B., 1994).

[6] Landsteiner K (1900). Zur Kenntnis der antifermentativen, lytischen und agglutinierenden Wirkungen des Blutserums und der Lymphe. Zentralblatt Bakteriologie.

[7] Edens C (2018). Evaluation of the National Healthcare Safety Network Hemovigilance Module for transfusion-related adverse reactions in the United States. Transfusion.

[8] Bernstein F (1924). Ergebnisse einer biostatischen zusammenfassenden Betrachtung uber die erblichen Blutstrukturen des Menschen. Klin Wochenschr.

[9] Svensson L (2013). Forssman expression on human erythrocytes: biochemical and genetic evidence of a new histo-blood group system. Blood.

[10] Hult AK, Olsson ML (2017). The FORS awakens: review of a blood group system reborn. Immunohematology.

[11] Schwarting GA, Kundu SK, Marcus DM (1979). Reaction of antibodies that cause paroxysmal cold hemoglobinuria (PCH) with globoside and Forssman glycosphingolipids. Blood.

[12] Kijimoto-Ochiai S, Takahashi W, Makita A (1981). Anti-Forssman antibody in human sera: properties and decreased level in cancer patients. Jpn J Exp Med.

[13] Jesus, C. *et al*. Prevalence of antibodies to a new histo-blood system: the FORS system. *Blood Transfus* 16, 178–183, 2016.0120-16 (2018). 10.2450/2016.0120-16 PMC5839615 27893352

[14] Hult AK, McSherry E, Moller M, Olsson ML (2018). GBGT1 is allelically diverse but dispensable in humans and naturally occurring anti-FORS1 shows an ABO-restricted pattern. Transfusion.

[15] Zhou D (2004). Lysosomal glycosphingolipid recognition by NKT cells. Science.

[16] Mattner, J. *et al*. Exogenous and endogenous glycolipid antigens activate NKT cells during microbial infections. *Nature* **434**, 525–529, nature03408 (2005). 10.1038/nature03408 15791258

[17] Wei, D. G., Curran, S. A., Savage, P. B., Teyton, L. & Bendelac, A. Mechanisms imposing the Vbeta bias of Valpha14 natural killer T cells and consequences for microbial glycolipid recognition. *J Exp Med* **203**, 1197–1207, jem.20060418 (2006). 10.1084/jem.20060418 PMC2121203 16651387

[18] Kronenberg, M. & Gapin, L. Natural killer T cells: know thyself. *Proc Natl Acad Sci USA* **104**, 5713–5714, 0701493104 (2007). 10.1073/pnas.0701493104 PMC1851556 17389403

[19] Speak, A. O. *et al*. Implications for invariant natural killer T cell ligands due to the restricted presence of isoglobotrihexosylceramide in mammals. *Proc Natl Acad Sci USA* **104**, 5971–5976, 0607285104 (2007). 10.1073/pnas.0607285104 PMC1851601 17372214

[20] Porubsky, S. *et al*. Normal development and function of invariant natural killer T cells in mice with isoglobotrihexosylceramide (iGb3) deficiency. *Proc Natl Acad Sci USA* **104**, 5977–5982, 0611139104 (2007). 10.1073/pnas.0611139104 PMC1851602 17372206

[21] Galili U, Rachmilewitz EA, Peleg A, Flechner I (1984). A unique natural human IgG antibody with anti-alpha-galactosyl specificity. J Exp Med.

[22] Tanemura M, Maruyama S, Galili U (2000). Differential expression of alpha-GAL epitopes (Galalpha1-3Galbeta1-4GlcNAc-R) on pig and mouse organs. Transplantation.

[23] Chung, C. H. *et al*. Cetuximab-induced anaphylaxis and IgE specific for galactose-alpha-1,3-galactose. *N Engl J Med* 358, 1109–1117, 358/11/1109 (2008). 10.1056/NEJMoa074943 PMC2361129 18337601

[24] Galili U (1989). Abnormal expression of alpha-galactosyl epitopes in man. A trigger for autoimmune processes?. Lancet.

[25] Yamamoto, F. *et al*. An integrative evolution theory of histo-blood group *ABO* and related genes. *Sci Rep* **4**, 6601, srep06601 (2014). 10.1038/srep06601 PMC5377540 25307962

[26] Xu H, Storch T, Yu M, Elliott SP, Haslam DB (1999). Characterization of the human Forssman synthetase gene. An evolving association between glycolipid synthesis and host-microbial interactions. J Biol Chem.

[27] Christiansen, D. *et al*. Humans lack iGb3 due to the absence of functional iGb3-synthase: implications for NKT cell development and transplantation. *PLoS Biol* **6**, e172, 07-PLBI-RA-3601 (2008). 10.1371/journal.pbio.0060172 PMC2459210 18630988

[28] Larsen RD, Rivera-Marrero CA, Ernst LK, Cummings RD, Lowe JB (1990). Frameshift and nonsense mutations in a human genomic sequence homologous to a murine UDP-Gal:beta-D-Gal(1,4)-D-GlcNAc alpha(1,3)-galactosyltransferase cDNA. J Biol Chem.

[29] Joziasse DH, Shaper JH, Jabs EW, Shaper NL (1991). Characterization of an alpha 1->3-galactosyltransferase homologue on human chromosome 12 that is organized as a processed pseudogene. J Biol Chem.

[30] Svensson, L. *et al*. The structural basis of blood group A-related glycolipids in an A3 red cell phenotype and a potential explanation to a serological phenomenon. *Glycobiology* **21**, 162–174, cwq143 (2011). 10.1093/glycob/cwq143 20926599

[31] Turcot-Dubois, A. L. *et al*. Long-term evolution of the CAZY glycosyltransferase 6 (ABO) gene family from fishes to mammals–a birth-and-death evolution model. *Glycobiology* **17**, 516–528, cwm016 (2007). 10.1093/glycob/cwm016 17298992

[32] Casals, F. *et al*. Human pseudogenes of the ABO family show a complex evolutionary dynamics and loss of function. *Glycobiology* **19**, 583–591, cwp017 (2009). 10.1093/glycob/cwp017 19218399

[33] Yamamoto F (2017). Evolutionary divergence of the *ABO* and *GBGT1* genes specifying the ABO and FORS blood group systems through chromosomal rearrangements. Sci Rep.

[34] Yamamoto F (1990). Cloning and characterization of DNA complementary to human UDP-GalNAc: Fuc alpha 1−>2Gal alpha 1−>3GalNAc transferase (histo-blood group A transferase) mRNA. J Biol Chem.

[35] Yamamoto F, Hakomori S (1990). Sugar-nucleotide donor specificity of histo-blood group A and B transferases is based on amino acid substitutions. J Biol Chem.

[36] Yamamoto F, McNeill PD (1996). Amino acid residue at codon 268 determines both activity and nucleotide-sugar donor substrate specificity of human histo-blood group A and B transferases: *In vitro* mutagenesis study. J Biol Chem.

[37] Seto NO (1997). Sequential interchange of four amino acids from blood group B to blood group A glycosyltransferase boosts catalytic activity and progressively modifies substrate recognition in human recombinant enzymes. J Biol Chem.

[38] Seto NO (1999). Donor substrate specificity of recombinant human blood group A, B and hybrid A/B glycosyltransferases expressed in *Escherichia coli*. Eur J Biochem.

[39] Boix E (2001). Structure of UDP complex of UDP-galactose:beta-galactoside-alpha −1,3-galactosyltransferase at 1.53-A resolution reveals a conformational change in the catalytically important C terminus. J Biol Chem.

[40] Gastinel LN (2001). Bovinealpha1,3-galactosyltransferase catalytic domain structure and its relationship with ABO histo-blood group and glycosphingolipid glycosyltransferases. EMBO J.

[41] Patenaude SI (2002). The structural basis for specificity in human ABO(H) blood group biosynthesis. Nat Struct Biol.

[42] Alfaro, J. A. *et al*. ABO(H) blood group A and B glycosyltransferases recognize substrate via specific conformational changes. *J Biol Chem* **283**, 10097–10108, M708669200 (2008). 10.1074/jbc.M708669200 18192272

[43] Heissigerova H, Breton C, Moravcova J, Imberty A (2003). Molecular modeling of glycosyltransferases involved in the biosynthesis of blood group A, blood group B, Forssman, and iGb3 antigens and their interaction with substrates. Glycobiology.

[44] Cid E, Yamamoto M, Buschbeck M, Yamamoto F (2013). Murine cell glycolipids customization by modular expression of glycosyltransferases. PLoS One.

[45] Cid, E., Yamamoto, M. & Yamamoto, F. Non-AUG start codons responsible for ABO weak blood group alleles on initiation mutant backgrounds. *Sci Rep* 7, 41720, srep41720 (2017). 10.1038/srep41720 PMC5282485 28139731

[46] Yamamoto M, Cid E, Yamamoto F (2017). Crosstalk between ABO and Forssman (FORS) blood group systems: FORS1 antigen synthesis by *ABO* gene-encoded glycosyltransferases. Sci Rep.

[47] Yamamoto M (2001). Murine equivalent of the human histo-blood group *ABO* gene is a *cis*-AB gene and encodes a glycosyltransferase with both A and B transferase activity. J Biol Chem.

[48] Schaefer AS (2010). A genome-wide association study identifies GLT6D1 as a susceptibility locus for periodontitis. Hum Mol Genet.

[49] Dupuy F (1999). A single amino acid in the hypervariable stem domain of vertebratealpha1,3/1,4-fucosyltransferases determines the type 1/type 2 transfer. Characterization of acceptor substrate specificity of the lewis enzyme by site-directed mutagenesis. J Biol Chem.

[50] Kaczmarek, R. *et al*. Human Gb3/CD77 synthase reveals specificity toward two or four different acceptors depending on amino acid at position 211, creating P(k), P1 and NOR blood group antigens. *Biochem Biophys Res Commun* **470**, 168–174, S0006-291X(16)30017-1 (2016). 10.1016/j.bbrc.2016.01.017 26773500

[51] Saitou N, Yamamoto F (1997). Evolution of primate ABO blood group genes and their homologous genes. Mol Biol Evol.

[52] Tamura, K. *et al*. MEGA5: molecular evolutionary genetics analysis using maximum likelihood, evolutionary distance, and maximum parsimony methods. *Mol Biol Evol* **28**, 2731–2739, msr121 (2011). 10.1093/molbev/msr121 PMC3203626 21546353

[53] Yamamoto, F., Yamamoto, M. & Blancher, A. Generation of histo-blood group B transferase by replacing the N-acetyl-D-galactosamine recognition domain of human A transferase with the galactose-recognition domain of evolutionarily related murinealpha1,3-galactosyltransferase. *Transfusion* **50**, 622–630, TRF2463 (2010). 10.1111/j.1537-2995.2009.02463.x 20042032

[54] Yamamoto M, Cid E, Yamamoto F (2017). ABO blood group A transferases catalyze the biosynthesis of FORS blood group FORS1 antigen upon deletion of exon 3 or 4. Blood Adv.

