# Supplementary material for: Amino acid substitutions at sugar-recognizing codons confer ABO blood group system-related α1,3 Gal(NAc) transferases with differential enzymatic activity
Source: Sci Rep. 2019 Jan 29;9:846. doi: 10.1038/s41598-018-37515-5 (PMC6351642; doi:10.1038/s41598-018-37515-5)
Supplement: Supplementary file 1 — Supplemental Figure 1 [file 41598_2018_37515_MOESM1_ESM.pdf]

Supplemental Information

Title:

Amino acid substitutions at sugar-recognizing codons confer ABO blood group system-related α1,3Gal(NAc) transferases with differential enzymatic activity

Authors:

Emili Cid[1,#], Miyako Yamamoto[1,#], and Fumiichiro Yamamoto[1,2,*]

[#]E.C. and M.Y. contributed equally to this work.

[*]To whom correspondence may be addressed.

ORCID identifiers: https://orcid.org/0000-0002-5025-352X (EC), https://orcid.org/0000-0001-9516-1402 (MY), https://orcid.org/0000-0001-9690-7034 (FY)

The α1,3-Gal(NAc) transferases are type II transmembrane proteins, having a single anchoring transmembrane domain flanked by a short N-terminal domain within the cytosol and a longer C-terminal domain in the Golgi lumen. Messenger RNAs encoding those enzymes may take several different splicing patterns. Therefore, the deduced amino acid sequences from human AT, mouse FS, rat iGb3S, and mouse GT cDNAs that were used to prepare the eukaryotic expression constructs were aligned. Results are shown below in **Supplemental Figure 1**.

```
                    #  *               #     #              #     #    #
H_ABO-A         MAEVLRTLAGK------------PKCHALRPMILFLIMLVLVLFGYGVLSPRSLMPGSLE     48
M_GBGT1         --MTRPRLAQG------------LAFFLLGGTGLWVLWKFIKDWLLVSYIP-YYLPCPEF     45
R_A3GALT2       --MALEGLRAKKRLLWR------LFLSAFGLLG-LYHYWFKIFRLFEVFIPMGICPMAIM     51
M_GGTA1         MITMLQDLHVNKISMSRSKSETSLPSSRSGSQEKIMNVKGKVILLMLIVSTVVVVFWEYV     60

                                            *           #*# *#* ***#####*##
H_ABO-A         RGFCMAVREPDHLQRVSLPRMVYPQPKVLTPCR-----KDVLVVTPWLAPIVWEGTFNID    103
M_GBGT1         FNMKLPFRKEKPLQPVT--QLQYPQPKLLEHGP-----TELLTLTPWLAPIVSEGTFDPE     98
R_A3GALT2       P----LLKDNFTG--------------VLRHWA----RPEVLTCTSWGAPIIWDETFDPH     89
M_GGTA1         NRTHSYQEDNVEGRREKGRNGDRIEEPQLWDWFNPKNRPDVLTVTPWKAPIVWEGTYDTA    120

                 #      ###*#*#****###*    #  **##*#  **#*  * #*#### # ##* *
H_ABO-A         ILNEQFRLQNTTIGLTVFAIKKYVA-FLKLFLETAEKHFMVGHRVHYYVFTDQPAAVPRV    162
M_GBGT1         LLKSMYQPLNLTIGVTVFAVGKYTC-FIQRFLESAEEFFMRGYQVHYYLFTHDPTAVPRV    157
R_A3GALT2       VAEREARRQNLTIGLTVFAVGRYLEKYLEHFLVSAEQYFMVGQNVVYYVFTDRPEAVPHV    149
M_GGTA1         LLEKYYATQKLTVGLTVFAVGKYIEHYLEDFLESADMYFMVGHRVIFYVMIDDTSRMPVV    180

                 *# ## * #   #  #**## ** ** ##          *#*###* ***  *    *
H_ABO-A         TLGTGRQLSVLEVRAYKRWQDVSMRRMEMISDFCERRFLSEVDYLVCVDVDMEFRDHVGV    222
M_GBGT1         PLGPGRLLSIIPIQGYSRWEEISMRRMETINKHIAKRAHKEVDYLFCVDVDMVFRNPWGP    217
R_A3GALT2       ALGQGRLLRVKPVRREKRWQDVSMARMLTLHEALGGQLGREADYVFCLDVDQYFSGNFGP    209
M_GGTA1         HLNPLHSLQVFEIRSEKRWQDISMMRMKTIGEHILAHIQHEVDFLFCMDVDQVFQDNFGV    240

                * *  *## ##       #  # ***#  * *     ******  # *** #     #*#
H_ABO-A         EILTPLFGTLHPGFYGSSREAFTYERRPQSQAYIPKDEGDFYYLGGFFGGSVQEVQRLTR    282
M_GBGT1         ETLGDLVAAIHPGYFAVPRRKFPYERRQVSSAFVADNEGDFYYGGALFGGRVARVYEFTR    277
R_A3GALT2       EVLADLVAQLHAWHFRWPRWMLPYERDKRSAAALSLSEGDFYYHAAVFGGSVAALLKLTA    269
M_GGTA1         ETLGQLVAQLQAWWYKASPEKFTYERRELSAAYIPFGEGDFYYHAAIFGGTPTHILNLTR    300

                 *      *    *#* *##*****# #   **#* ****# *#      #      #
H_ABO-A         ACHQAMMVDQANGIEAVWHDESHLNKYLLRHKPTKVLSPEYLWDQQLLGWPAVLRKLRFT    342
M_GBGT1         ACHMAILADKANSIMAAWQEESHLNRHFIWHKPSKVLSPEYLWDERKP-RPRSLKMIRFS    336
R_A3GALT2       HCATGQQLDREHGIEARWHDESHLNKFFWLSKPTKLLSPEFCWAEEIG-WRPEIHHPRLI    328
M_GGTA1         ECFKGILQDKKHDIEAQWHDESHLNKYFLFNKPTKILSPEYCWDYQIG-LPSDIKSVKVA    359

                   *    #*
H_ABO-A         AVPKNHQAVRNP*-                                                  354
M_GBGT1         SVKKNANWLRT*--                                                  347
R_A3GALT2       WAPKEYALVRT*--                                                  339
M_GGTA1         WQTKEYNLVRNNV*                                                  372


* 4 identical
# 3 identical
```

Supplemental Figure 1. Amino acid sequence comparison among α1,3-Gal(NAc) transferases. The deduced amino acid sequences of human AT, mouse FS, rat iGb3S, and mouse GT cDNAs used in the present study were aligned, using the ClustralW program of MEGA5 software. They were numbered as shown on the right sides of the sequences. Amino acid sequences in alternate exons are shown in blue and black colors, whereas those at the splicing junctions are shown in red. The crucial amino acid residues corresponding to codons 266 to 268 of human AT and BT are indicated in bold type and yellow highlighted. When amino acids are conserved in all the four transferases, they are marked above with a star symbol (*), whereas they are marked above with the (#) symbol when they

are conserved in three of the four proteins. Sequence homology was observed mainly in the C-terminal domains of those α1,3-Gal(NAc) transferases as previously reported. The sequences are more conserved in the last two coding exons among four transferases, whereas the last three exons are conserved between AT and FS. Approximately 53% of amino acids are maintained in at least three proteins in the last two coding exons. In addition to protein sequences, homology is also observed in the nucleotide sequences and the exon-intron organization of those genes. *ABO* and *GBGT1* genes are more homologous to each other than to *A3GALT2* or *GGTA1* genes, and the *A3GALT2* and *GGTA1* genes are more homologous to one another than to *ABO* or *GBGT1*, as shown in the Ensembl gene tree ENSGT00400000022032 (data not shown).
